# NOD2 protects against allergic lung inflammation in obese female mice

**DOI:** 10.1016/j.isci.2024.111130

**Published:** 2024-10-11

**Authors:** Rodrigo Rodrigues e-Lacerda, Nicole G. Barra, Han Fang, Gabriel Forato Anhê, Jonathan D. Schertzer

**Affiliations:** 1 Department of Biochemistry and Biomedical Sciences, Farncombe Family Digestive Health Research Institute, Centre for Metabolism, Obesity and Diabetes Research, McMaster University, 1200 Main Street West, Hamilton, ON L8N 3Z5, Canada; 2 Department of Translational Medicine, University of Campinas, Rua Tessália Vieira de Camargo, 126, Cidade Universitária Zeferino Vaz, Campinas, SP CEP 13083-887, Brazil

**Keywords:** pathophysiology, immunology, cell biology

## Abstract

Obesity is associated with compartmentalized changes in immune responses that can be protective or pathogenic. It has been proposed that obesity-related changes in the microbiota influence allergic lung inflammation. We hypothesized that sensors of the bacterial cell wall influenced allergenic lung inflammation during obesity. Ovalbumin (OVA)-induced lung inflammation was similar in female Nod1^−/−^ and wild-type mice during high-fat-diet-induced obesity, but allergic lung inflammation was higher in obese, high-fat-diet-fed female Nod2^−/−^ mice. Obese Nod2^−/−^ mice had higher inflammatory cell infiltration in the bronchial alveolar lavage (BAL) and lungs, pulmonary fibrosis, mucus levels, hypertrophy and hyperplasia of goblet cells, M2 alveolar macrophage infiltration, interleukin-4 (IL-4), IL-5, IL-6, and lower CXCL1 and IL-22. Therefore, Nod2 protects against excessive lung inflammation and is a bacterial sensor that relays protective responses to allergenic lung inflammation in obese female mice.

## Highlights

• Obesity alters immune responses, which can either protect or worsen lung inflammation
• Obesity does not modify lung inflammation in Nod1^−/−^ mice
• Obese Nod2^−/−^ mice had higher lung inflammation, fibrosis, and mucus production
• Nod2 relays protective immune responses to reduce allergic lung inflammation

## Graphical abstract

**Figure:**
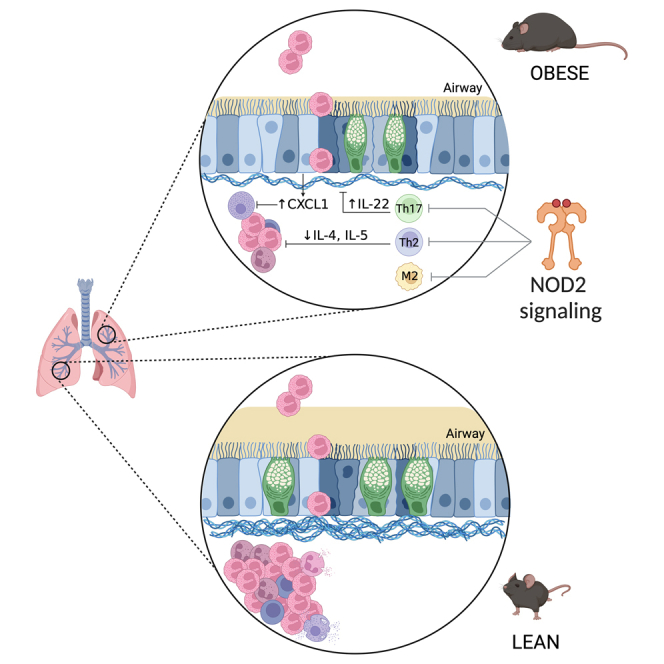

## Introduction

Although obesity has been associated with chronic, systemic inflammation, this does not accurately reflect the local immune status in specific tissues. Obesity is associated with compartmentalized changes in immune responses.^1^ For example, diet-induced obesity can lower immune responses in the upper intestine but increase inflammation in adipose tissue and the liver.^2^ The intestinal microbiota is positioned to influence compartmentalized immune responses where symbiotic relationships in the microbiota that are altered by aspects of obesity can generate protective or pathogenic immunometabolism responses in different tissues.^3^ For example, obesity protects against allergic lung inflammation in allergen-sensitized female mice.^4^

Allergic asthma is a chronic inflammatory disease characterized by an immune-mediated hypersensitivity reaction induced primarily by exposure to airborne allergens.^5^ The inflammatory response during allergenic asthma is initiated by type 2 T lymphocytes (Th2),^6^ which is propagated by eosinophils, and by humoral immunity, through the production of immunoglobulin E (IgE).^7^ The production of different Th2 cytokines in response to allergens promotes allergic pathology. For example, interleukin-4 (IL-4) and IL-13 stimulate B lymphocytes to produce IgE and induce smooth muscle contraction responsible for bronchospasm and lung remodeling via hypertrophy and hyperplasia of mucus-producing goblet cells promoting pulmonary fibrosis. IL-5 is responsible for stimulating the survival, maturation, and activation of the allergic asthma effector cell, the eosinophil.^8^ Eosinophils produce a variety of asthma-inducing factors including major basic protein (MBP), eosinophilic peroxidase (EPO), eosinophil cationic protein (ECP), leukotrienes, and remodeling factors, such as transforming growth factor beta (TGF-β), IL-13, matrix metalloproteinase (MMP), vascular endothelial growth factor (VEGF), basic fibroblast growth factor (bFGF), and nerve growth factor (NGF).^9^

Allergic airway disease is characterized by persistent and recurrent wheezing, coughing, and difficulty breathing, associated with reversible airflow obstruction and airway hyperresponsiveness.^10^ Despite having a low mortality rate, asthma affects millions of people, causing a burden on individuals and health systems through treatments that last a lifetime.^11^ Clinical studies indicate that obesity and overweight status are important risk factors responsible for modulating the development and progression of allergic airway disease.^12^^,^^13^ Diets high in saturated fat, in addition to promoting obesity, lead to changes in the composition of microbiome,^13^ but the role of the host-microbe relationship in the pathophysiology of asthma remains poorly understood.

The lower respiratory tract (LTR) and intestine are key mucosal barriers that harbor many bacteria.^14^^,^^15^ There is a bidirectional connection between the lungs and intestine, as lung diseases can be influenced by the intestinal microbiota and vice versa.^16^ Asthmatic children with high lung levels of IL-4, IL-5, and IL-13 have intestinal microbiota rich in bacteria from the genus *Clostridium* and *Bacteroides*, whereas healthy children with low levels of these interleukins are colonized by *Lachnospira*, *Rothia*, *Veillonella,* and *Lactobacillaceae*.^17^ Rather than correlate microbiome taxonomy with outcomes, one key problem is to identify the bacterial sensors of the host immune system that relay changes in allergic lung inflammation.

The nucleotide-binding oligomerization domain (NOD)-like receptors (NLRs) NOD1 and NOD2 are bacterial sensors that recognize bacterial cell wall muropeptides diaminopimelic acid dipeptide (iEDAP) and muramyl dipeptide (MDP), respectively. NOD1 and NOD2 activate nuclear factor κB (NF-κB) and mitogen-activated protein kinase (MAPK) pathways, stimulating the transcription of pro-inflammatory cytokines and antimicrobial peptides.^18^ NOD receptors are present in different cell types, but higher levels of NOD1 are detected in epithelial and endothelial cells, in contrast to NOD2, which is more prominent in the hematopoietic compartment.^19^ Eosinophils express NOD1 and NOD2.^9^ Despite some similar signaling pathways, NOD1 and NOD2 can produce divergent metabolic and immunometabolism outcomes, including regulation of blood glucose and adipose tissue inflammation during obesity.^20^^,^^21^^,^^22^^,^^23^ NOD1 and NOD2 can balance pathogenic and protective immune responses during obesity.^24^ NOD2 in non-hematopoietic cells protected mice from obesity-induced metabolic inflammation and insulin resistance by limiting bacterial infiltration into metabolic tissues.^21^ The NOD2 ligand, MDP, reduced obesity and lipopolysaccharide (LPS)-induced inflammation in a mechanism independent of the adapter protein RIPK2.^23^ This has fostered the concept that NOD2 may provide immune tolerization effects to dampen inflammation, but still, it is not clear how NOD1 or NOD2 influences allergic lung inflammation during obesity.

We have previously demonstrated that obesity alters allergic lung inflammation in a sex-specific manner where obese female mice have reduced expression of NF-κB and leukocyte adhesion proteins.^4^ Subsequently, it was shown that deletion of NOD1 or its downstream adapter RIPK2, but not NOD2, in female mice improved features of asthma.^25^ Therefore, NOD1 and NOD2 can have opposing roles in aspects of inflammatory lung disease, but it was not clear if NOD1 or NOD2 influenced the protective effect of obesity on allergenic lung inflammation. In the present study, we show that attenuation of the inflammatory response observed in obese female mice requires NOD2 in a model of allergenic airway disease.

## Results

### Deletion of NOD1 or NOD2 alters metabolism in high-fat diet-fed mice

**Figure 1:**
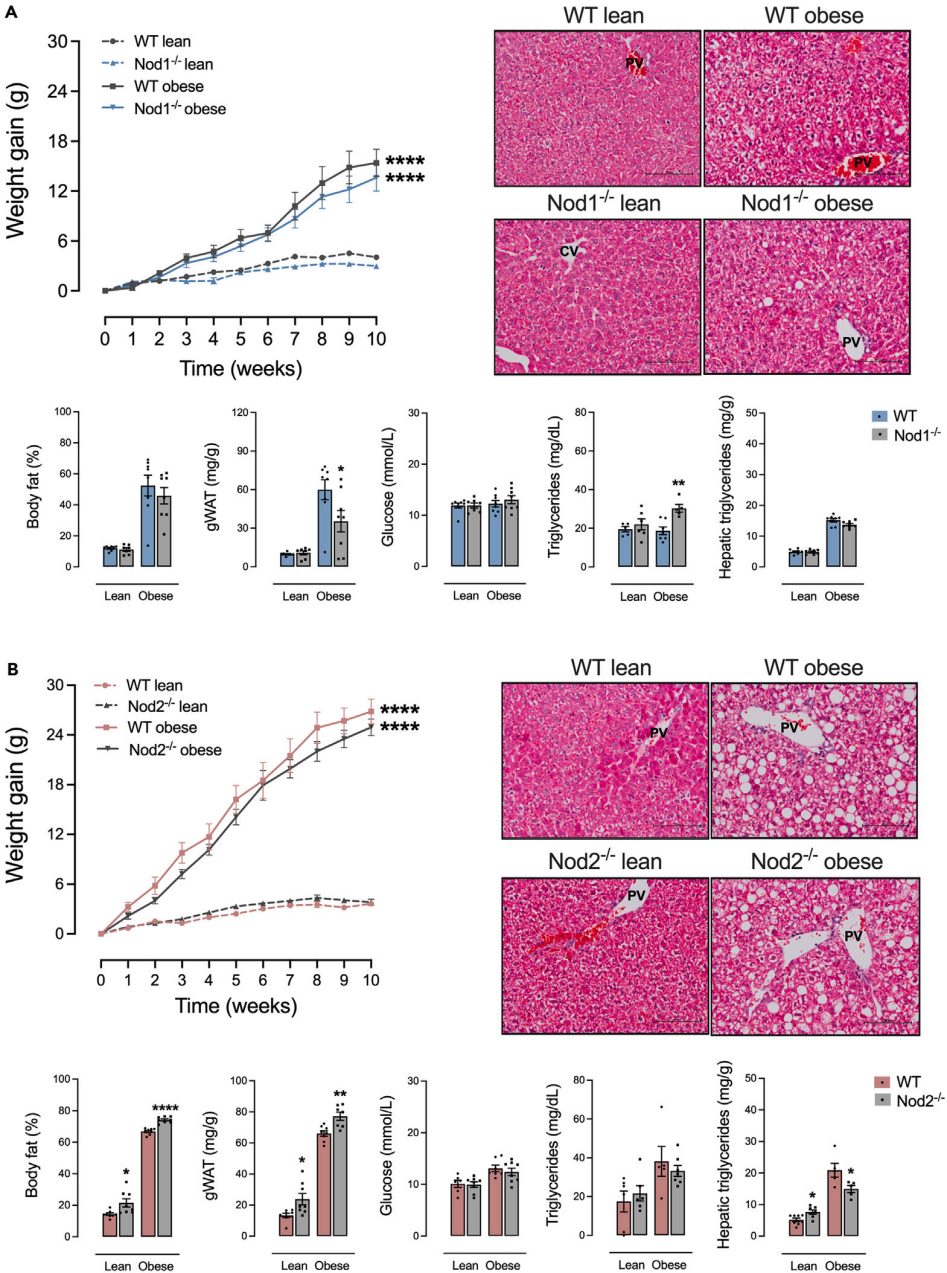
NOD1 or NOD2 deficiency influences metabolism in HFD-fed mice (A and B) Weight gain over 10 weeks, body fat percentage, gonadal adipose tissue mass, random-fed blood glucose, serum triglycerides, hepatic triglycerides, and a representative histological image of the liver (stained with H&E) in (A) Nod1^−/−^ versus C57Bl/6N (WT) and (B) Nod2^−/−^ versus C57Bl/6J (WT) mice fed a standard control or HFD. Results are shown as mean ± SEM. The results were analyzed by the ROUT test with Q = 1% to exclude outliers. Statistical differences were identified by Unpaired Student’s t test. *p* values refer to comparisons between WT mice versus Nod mice. ∗*p* < 0.05; ∗∗*p* < 0.01; ∗∗∗*p* < 0.001, and ∗∗∗∗*p* < 0.0001. n = 5–8. WT, wild type; PV, portal vein; CV, central vein. Scale bar, 100 μM.

Compared to a control diet, feeding a high-fat diet (HFD) increased body weight gain, body fat percentage, and hepatic steatosis to a similar extent in female Nod1^−/−^ and wild-type (WT) mice. However, HFD-fed Nod1^−/−^ mice had lower gonadal white adipose tissue (gWAT) mass and higher serum levels of triglycerides compared to HFD-fed WT mice. No differences were detected in random-fed blood glucose between Nod1^−/−^ and WT mice (Figure 1A). Compared to a control diet, an HFD increased body weight gain, serum glucose, and serum triglycerides to a similar extent in female Nod2^−/−^ and WT mice. HFD-fed Nod2^−/−^ mice had higher body fat percentage and gWAT mass but lower hepatic steatosis compared to HFD-fed WT mice. Control-diet-fed Nod2^−/−^ had a similar phenotype, but with higher steatosis compared to control-diet-fed WT mice (Figure 1B).

### Deletion of NOD2 exacerbates BAL leukocyte infiltrate during allergic lung inflammation

**Figure 2:**
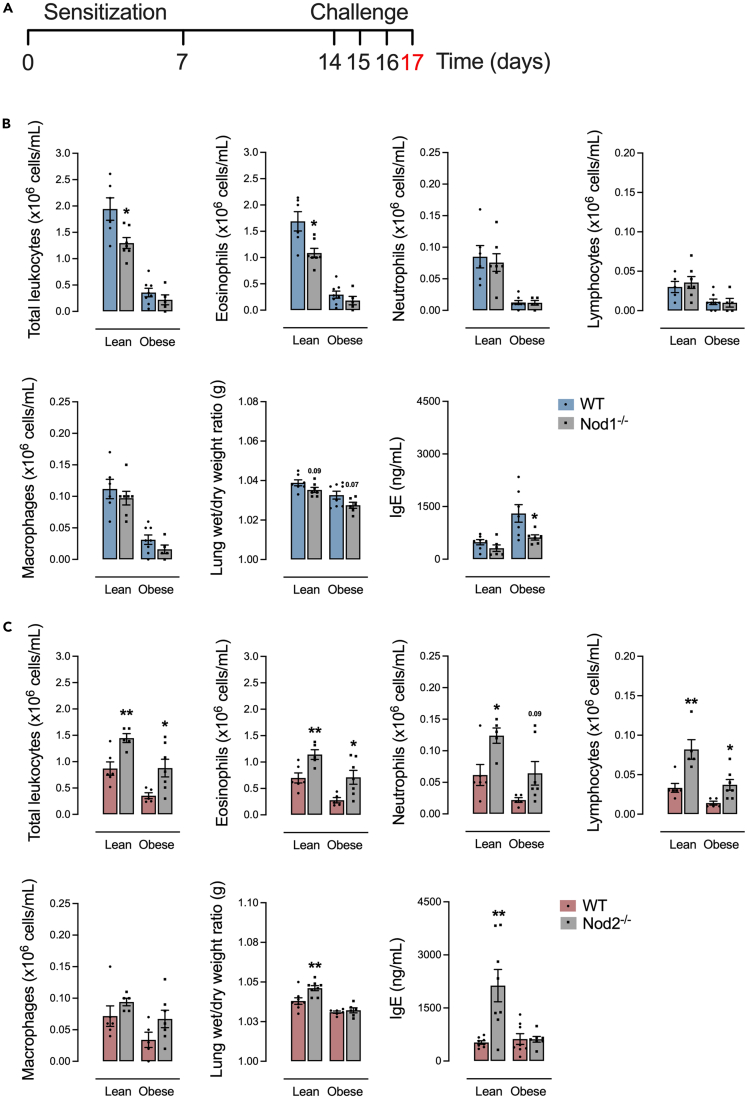
BAL leukocyte infiltrate is aggravated by NOD2 deletion during obesity and allergic lung inflammation (A) Protocol of allergic lung inflammation. (B and C) Total leukocytes, eosinophils, neutrophils, lymphocytes, and macrophages in BAL, pulmonary edema, and serum levels of IgE in (B) Nod1^−/−^ versus C57Bl/6N (WT) and (C) Nod2^−/−^ versus C57Bl/6J (WT) mice fed a standard control or HFD. Results are shown as mean ± SEM. The results were analyzed by the ROUT test with Q = 1% to exclude outliers. Statistical differences were identified by Unpaired Student’s t test. *p* values refer to comparisons between WT mice versus Nod mice. ∗*p* < 0.05; ∗∗*p* < 0.01; ∗∗∗*p* < 0.001, and ∗∗∗∗*p* < 0.0001. n = 5–8. WT, wild type.

In response to the allergic lung inflammation model (Figure 2A), control-diet-fed Nod1^−/−^ mice had lower total leukocytes, mainly eosinophils, compared to control-diet-fed WT mice. HFD-fed Nod1^−/−^ mice had lower IgE compared to WT mice. No differences were detected in the number of neutrophils, lymphocytes, macrophages, or pulmonary edema, regardless of diet in Nod1^−/−^ versus WT mice (Figure 2B). Control-diet-fed Nod2^−/−^ mice had higher total leukocytes, including eosinophils, neutrophils, and lymphocytes, and higher pulmonary edema and serum IgE compared to control-diet-fed WT mice. HFD-fed Nod2^−/−^ mice had higher total leukocytes, mainly eosinophils, and lymphocytes, in BAL compared to HFD-fed WT mice. No differences were detected in the number of macrophages, regardless of diet in Nod2^−/−^ versus WT mice (Figure 2C).

### Deletion of NOD2, but not NOD1, promotes lung inflammation

**Figure 3:**
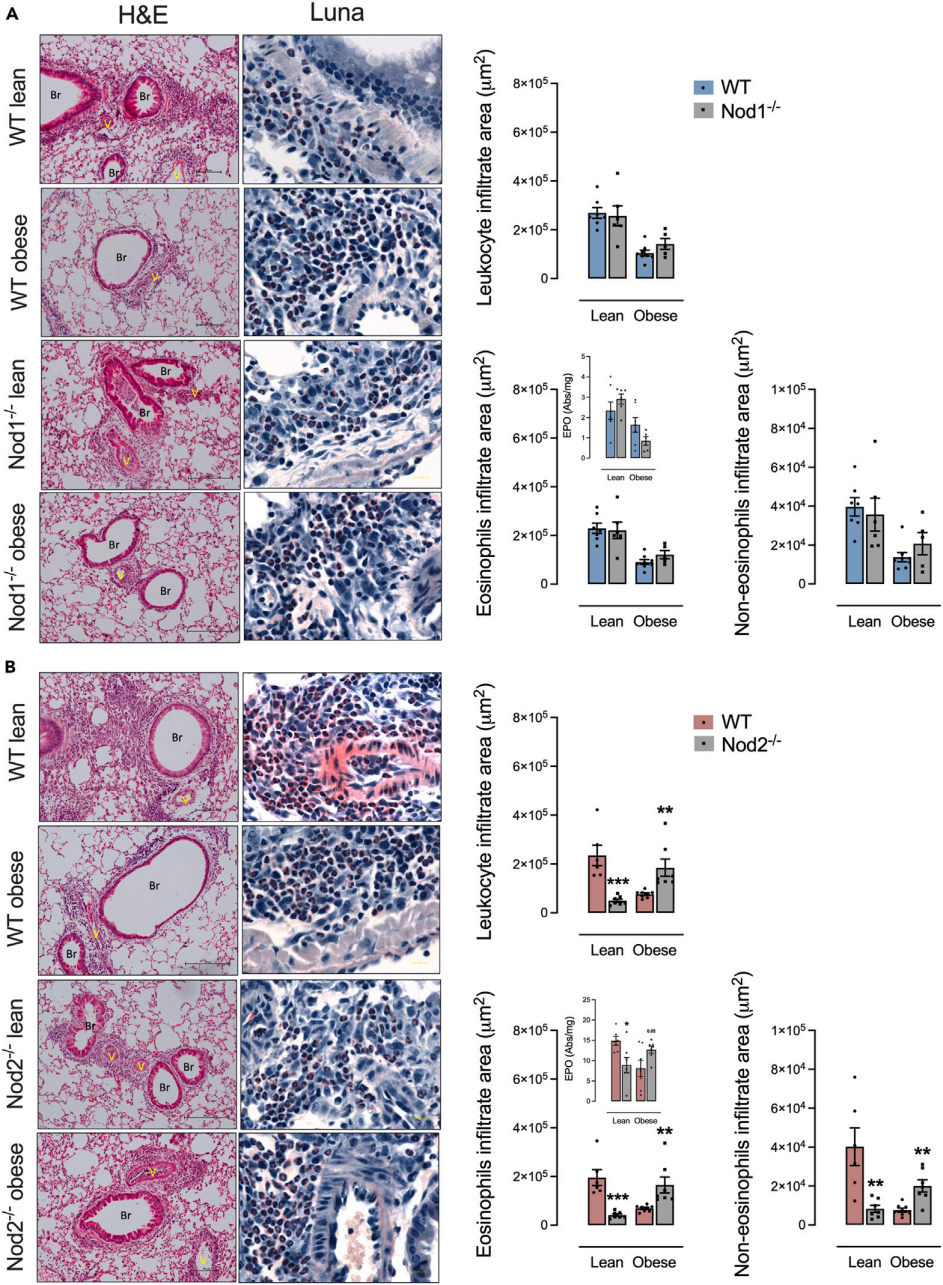
Lung leukocyte infiltrate is aggravated by NOD2 deletion during obesity and allergic lung inflammation (A and B) Lung inflammation in (A) Nod1^−/−^ versus C57Bl/6N (WT) mice and (B) Nod2^−/−^ versus C57Bl/6J (WT) mice fed a standard control or HFD measured by leukocyte infiltrate in the peri-bronchoalveolar space, as well as the area derived from the relative count of eosinophils, non-eosinophils, and the eosinophilic peroxidase (EPO) enzymatic activity determination. Histological panels of the lung were stained by H&E (left) and Luna staining (right, showing eosinophils marked in red) showing infiltration of leukocytes (mainly eosinophils) in the peri-bronchoalveolar and perivascular space. Results are shown as mean ± SEM. The results were analyzed by the ROUT test with Q = 1% to exclude outliers. Statistical differences were identified by Unpaired Student’s t test. *p* values refer to comparisons between WT mice versus Nod mice. ∗*p* < 0.05; ∗∗*p* < 0.01; ∗∗∗*p* < 0.001, and ∗∗∗∗*p* < 0.0001. n = 5–8. WT, wild type; Br, bronchiole; V, vessel. Eosinophils stained in red by Luna stain. Scale bar, 100 μM.

HFD-fed Nod1^−/−^ and HFD-fed WT mice had similar leukocyte infiltration and EPO levels in the lung parenchyma, effects that were also seen in control-diet-fed Nod1^−/−^ and WT mice, indicating that NOD1 did not alter these inflammatory indicators of lung inflammation, regardless of diet (Figure 3A). Nod2^−/−^ mice fed HFD showed higher eosinophilic and non-eosinophilic infiltrate and EPO levels in the lung compared to HFD-fed WT mice (Figure 3B). Control-diet-fed Nod2^−/−^ mice had lower infiltrate of eosinophils, non-eosinophils, and EPO levels compared to control-diet-fed WT (Figure 3B). Therefore, the deletion of NOD2, but not NOD1, increased inflammation in the lung parenchyma during obesity.

### Deletion of NOD2 exacerbates lung fibrosis during obesity

**Figure 4:**
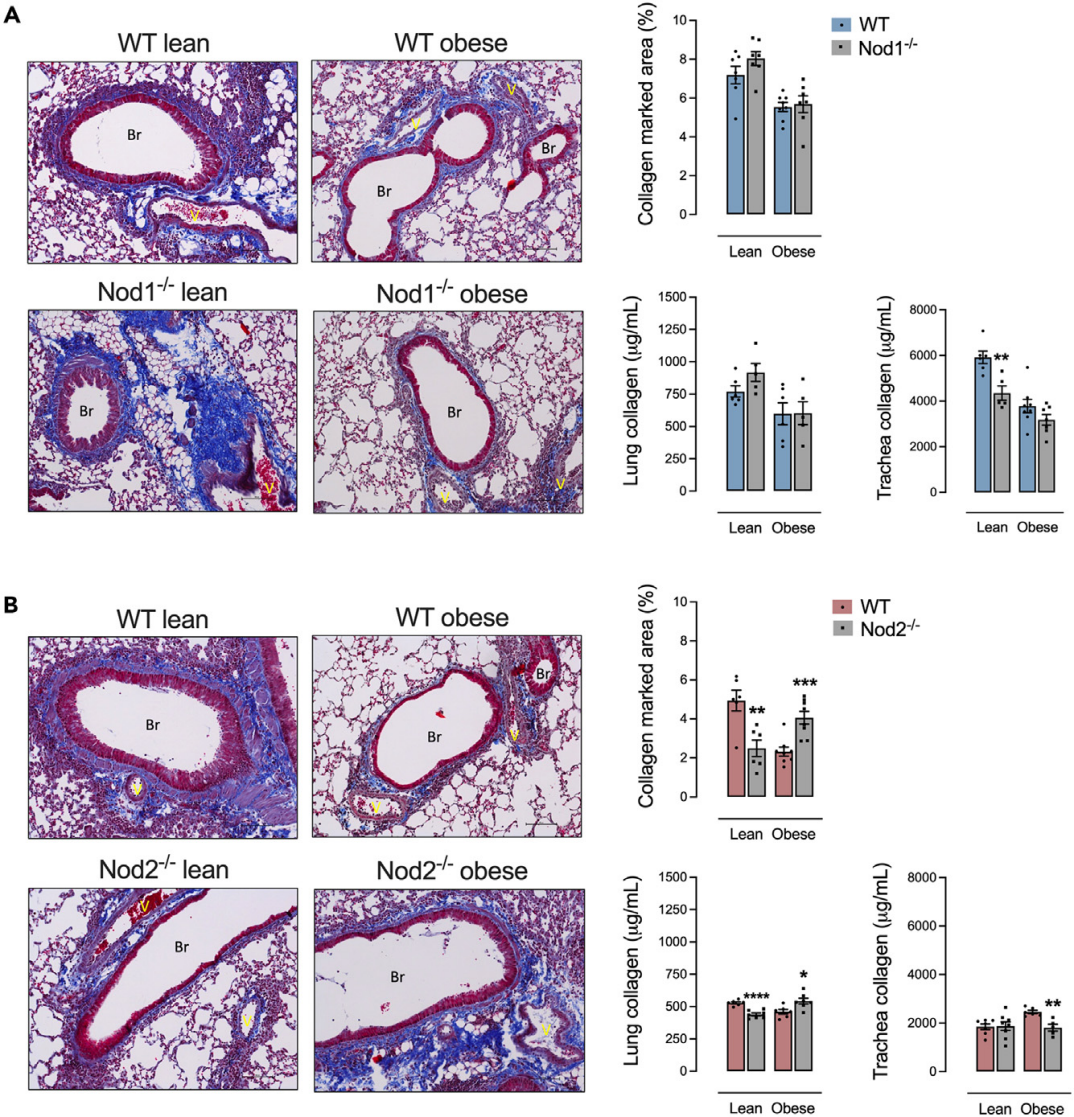
NOD2 deletion worsens lung fibrosis during obesity and allergic lung inflammation (A and B) Lung fibrosis in (A) Nod1^−/−^ versus C57Bl/6N (WT) mice and (B) Nod2^−/−^ versus C57Bl/6J (WT) mice fed a standard control or HFD measured by collagen marked area in lung stained by Masson’s trichrome, where it is possible to observe collagen fibers in dark blue surrounding the peri-bronchoalveolar and perivascular space. Quantification of collagen levels in lung and trachea was also determined by hydroxyproline measurement using a collagen curve obtained from bovine tendon as standard. Results are shown as mean ± SEM. The results were analyzed by the ROUT test with Q = 1% to exclude outliers. Statistical differences were identified by unpaired Student’s t test. *p* values refer to comparisons between WT mice versus Nod mice. ∗*p* < 0.05; ∗∗*p* < 0.01; ∗∗∗*p* < 0.001, and ∗∗∗∗*p* < 0.0001. n = 5–8. WT, wild type; Br, bronchiole; V, vessel. Collagens fibers stained in dark blue by Masson’s trichrome stain. Scale bar, 100 μM.

Control-diet-fed Nod1^−/−^ mice had lowered tracheal stenosis compared to control-diet-fed WT. No differences were detected in the levels of collagen, regardless of diet in Nod1^−/−^ versus WT mice (Figure 4A). Control-diet-fed Nod2^−/−^ had lower levels of collagen in the lung. HFD-fed Nod2^−/−^ mice had higher levels of collagen in the lung and lower tracheal stenosis compared to HFD-fed WT mice (Figure 4B).

### Deletion of NOD2 promotes lung goblet cell hypertrophy and mucus content during obesity

**Figure 5:**
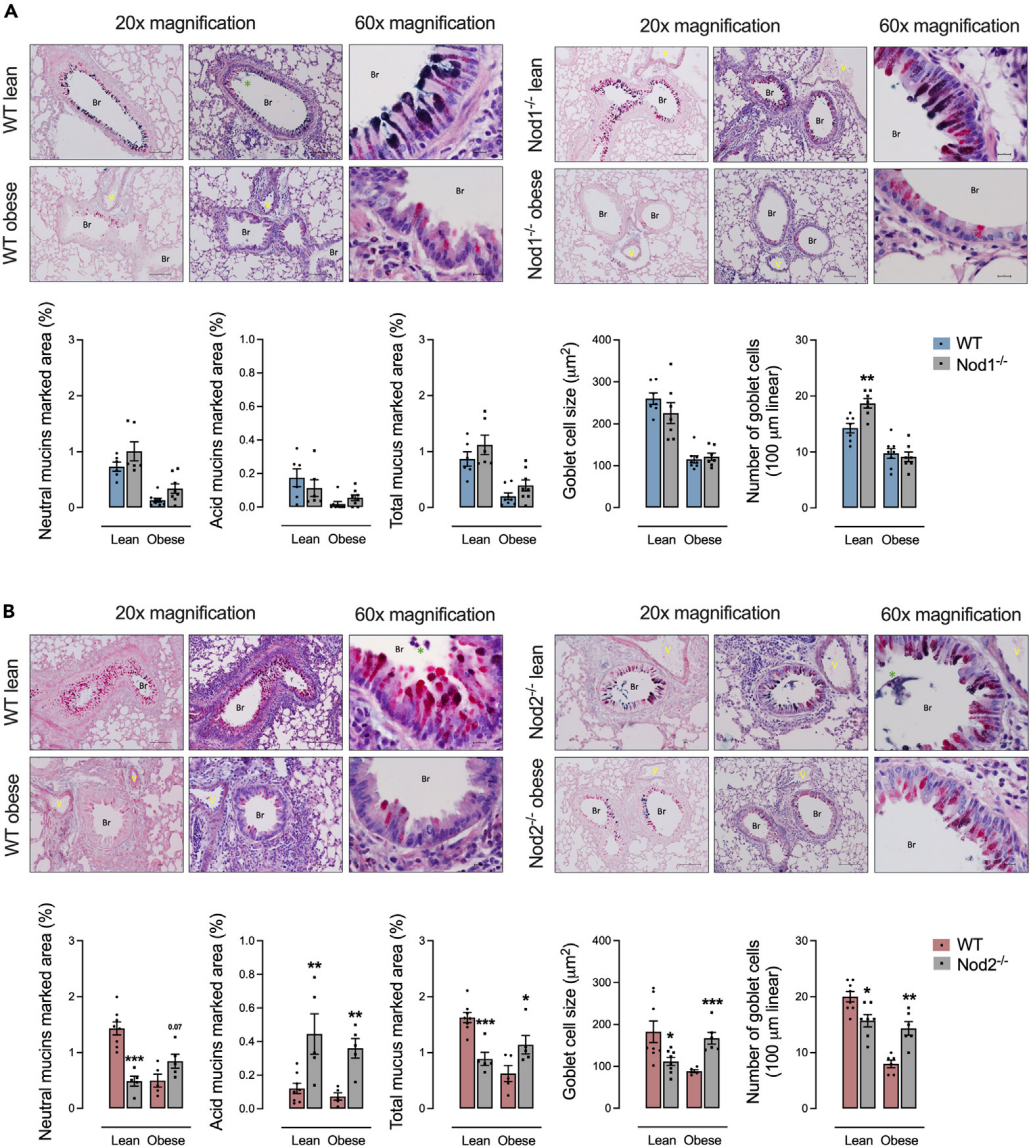
NOD2 deletion enhances lung goblet cell hypertrophy and mucus production during obesity and allergic lung inflammation (A and B) Lung goblet cells and mucins in (A) Nod1^−/−^ versus C57Bl/6N (WT) mice and (B) Nod2^−/−^ versus C57Bl/6J (WT) mice fed a standard control or HFD measured by neutral mucus (in purple-magenta), acidic mucus (in turquoise blue), total mucus, and goblet cell size and number shown in lung tissue stained by the association of Schiff’s periodic acid (for neutral mucins) and Alcian blue (for acid mucins). Results are shown as mean ± SEM. The results were analyzed by the ROUT test with Q = 1% to exclude outliers. Statistical differences were identified by unpaired Student’s t test. *p* values refer to comparisons between WT mice versus Nod mice. ∗*p* < 0.05; ∗∗*p* < 0.01; ∗∗∗*p* < 0.001, and ∗∗∗∗*p* < 0.0001. n = 5–8. WT, wild type; Br, bronchiole; V, vessel. Green asterisk = mucus plugs. Neutral mucus stained in purple-magenta by periodic acid-Schiff and acid mucus stained in turquoise blue by Alcian blue. Scale bar, 100 μM.

In mice fed a control diet, Nod1^−/−^ mice had a higher number of goblet cells compared to control diet-fed WT mice. No differences were detected in the levels of neutral and acid mucins and goblet cell size, regardless of diet in Nod1^−/−^ versus WT mice (Figure 5A). HFD-fed Nod2^−/−^ mice had higher mucus production, mainly acidic mucins, and goblet cell hypertrophy and hyperplasia compared to obese HFD-fed WT mice. Control-diet-fed Nod2^−/−^ mice had lower mucus production, mainly neutral mucins, and lower size and number of goblet cells compared to control-diet-fed WT mice (Figure 5B).

### Deletion of NOD2 increases M2 macrophage polarization during obesity and allergic lung inflammation

**Figure 6:**
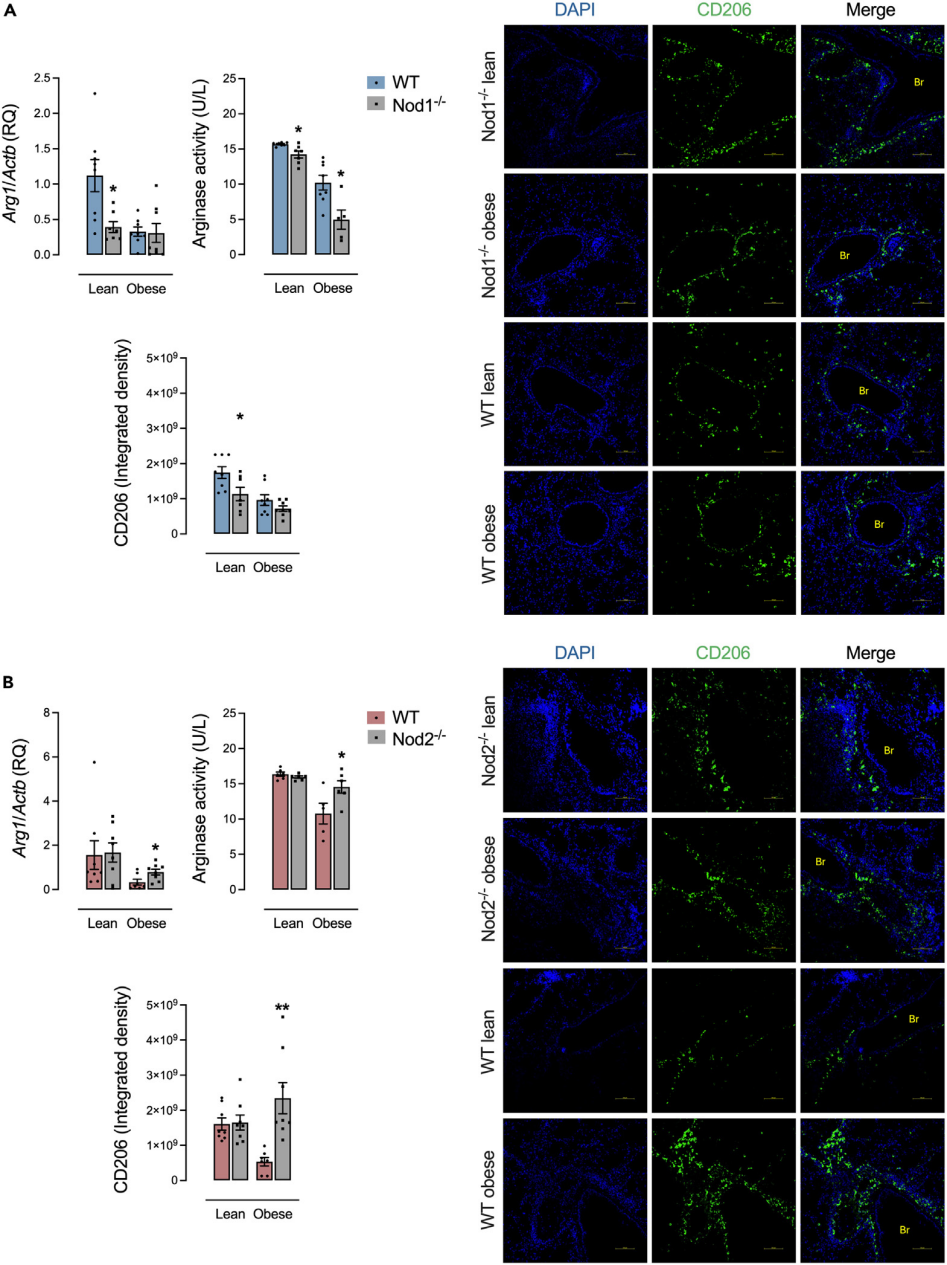
M2 macrophage polarization is increased by NOD2 deletion during obesity and allergic lung inflammation (A and B) M2 macrophage characterization in (A) Nod1^−/−^ versus C57Bl/6N (WT) mice and (B) Nod2^−/−^ versus C57Bl/6J (WT) mice fed a standard control or HFD measured by the lung gene expression of the enzyme arginase-1 (*Arg1*), arginase activity in the BAL, and lung expression of CD206. Results are shown as mean ± SEM. The results were analyzed by the ROUT test with Q = 1% to exclude outliers. Statistical differences were identified by unpaired Student’s t test. *p* values refer to comparisons between WT mice versus Nod mice. ∗*p* < 0.05; ∗∗*p* < 0.01; ∗∗∗*p* < 0.001, and ∗∗∗∗*p* < 0.0001. n = 5–8. WT, wild type; Br, bronchiole. Scale bar, 100 μM.

Control-diet-fed Nod1^−/−^ mice had lower lung expression of *Arg1*, arginase activity in the BAL, and lung expression of CD206 compared to control diet-fed WT mice. During obesity, Nod1^−/−^ mice had just lower arginase activity in the BAL (Figure 6A). HFD-fed Nod2^−/−^ mice had higher lung expression of *Arg1*, arginase activity in the BAL, and lung expression of CD206 compared to HFD-fed WT mice. No differences were detected in Nod2^−/−^ versus WT control-diet-fed mice (Figure 6B).

### Deletion of NOD2 modifies the secretion of allergic response cytokines and chemokines during obesity and allergic lung inflammation

**Figure 7:**
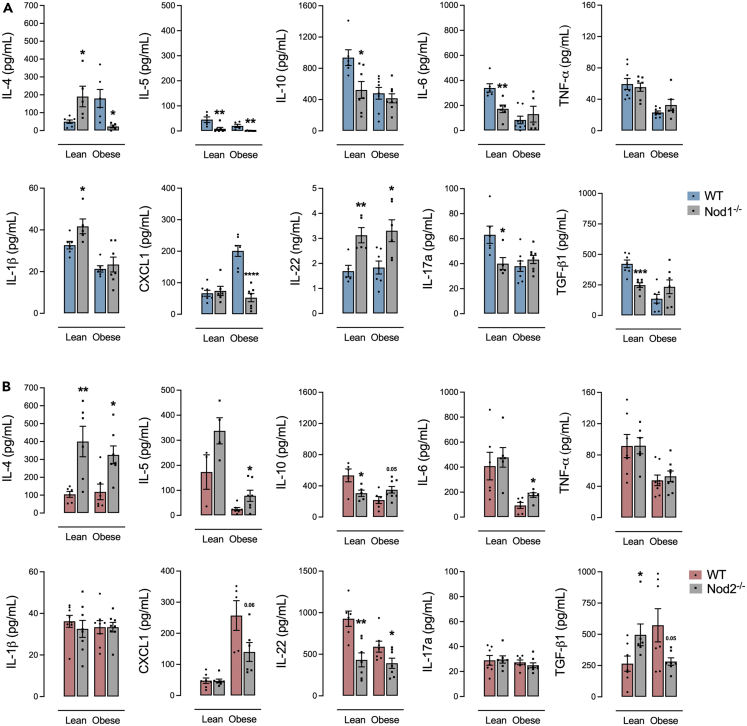
NOD1 or NOD2 deletion affects the secretion of allergic response cytokines and chemokines during allergic lung inflammation (A and B) BAL levels of IL-4, IL-5, IL-10, IL-6, TNF-α, IL-1β, CXCL1, IL-22, IL-17a, and TGF-β1 in (A) Nod1^−/−^ versus C57Bl/6N (WT) mice and (B) Nod2^−/−^ versus C57Bl/6J (WT) mice fed a standard control or HFD. Results are shown as mean ± SEM. The results were analyzed by the ROUT test with Q = 1% to exclude outliers. Statistical differences were identified by unpaired Student’s t test. *p* values refer to comparisons between WT mice versus Nod mice. ∗*p* < 0.05; ∗∗*p* < 0.01; ∗∗∗*p* < 0.001, and ∗∗∗∗*p* < 0.0001. n = 5–8. WT, wild type.

HFD-fed Nod1^−/−^ mice had lower IL-4, IL-5, and CXCL1 and higher IL-22 in BAL compared to HFD-fed WT mice. Control-diet-fed Nod1^−/−^ mice had lower IL-5, IL-10, IL-6, IL-17a, and TGF-β1 and higher IL-4, IL-1β, and IL-22 in BAL compared to control-diet-fed WT mice (Figure 7A). HFD-fed Nod2^−/−^ mice had lower CXCL1 and IL-22 and higher IL-4, IL-5, and IL-6 compared to HFD-fed WT mice. Control-diet-fed Nod2^−/−^ mice had lower IL-10 and IL-22 and higher IL-4 and TGF-β1 compared to control-diet-fed WT mice (Figure 7B).

## Discussion

We previously demonstrated that diet-induced obese female mice have lower inflammatory lung infiltration in an ovalbumin (OVA)-induced allergic lung inflammation model.^4^ In the present study, we show that the bacterial sensor NOD2 is required for lower inflammatory lung infiltration in obese female mice. Yahia (2021) found that deletion of NOD1 or RIPK2, but not NOD2, inhibited both BAL inflammation and airway responsiveness in response to methacholine in house dust mite (HDM)-induced allergic asthma in mice.^25^ Using OVA, we corroborate that deletion of NOD1 in lean mice lowers inflammation, mainly in the BAL. However, NOD1 had little or no role in altering lung inflammation during diet-induced obesity. We provide evidence that the deletion of NOD2 enhanced lung inflammation and is required for dampening lung inflammation during diet-induced obesity in female mice. Hence, we have shown that diet or obesity can be a factor that dictates the role of NODs in several aspects of allergic lung inflammation. Differences in allergic inflammation susceptibility in RIPK2-deficient mice have been reported in OVA and HDM models,^26^^,^^27^ suggesting that the choice of the allergenic agent is an important factor. One possible explanation is the route of exposure to these allergens since OVA was administered by aerosol here compared to HDM, a purified allergenic extract from *Dermatophagoides farina*, administered intranasally. Another possible explanation is the purity of the immunogenic agent. Commercially available HDM extracts usually have variable endotoxin (LPS) levels in their composition, ranging from 31 to 52,000 units, which may define a different immunological phenotype due to the concomitant activation of TLR4.^28^^,^^29^ It is also known that one of the major HDM components, the Der p 2 protein, acts as an MD2-like chaperonin that promotes TLR4 signaling in airway structural cells, which further expands the Th1 response of this preparation.^30^

Obesity reduces lung remodeling during OVA exposure; however, NOD2 attenuates the structural pathological lung changes caused by allergic airway inflammation in female mice since obese NOD2-deficient mice display enhanced tissue damage induced by OVA. In addition to BAL, a detailed evaluation of lung tissue is essential for understanding the outcomes of allergic asthma. It is known that obesity alters the transition of inflammatory cells through the airway epithelium to the lumen, modifying the expression of adhesion molecules such as the epithelial cell adhesion molecule (EpCAM) or the integrin CD103,^4^^,^^31^ which contributes to damage to the lung parenchyma through prolonged exposure to inflammatory factors secreted by these cells. Yahia (2021) showed that despite a reduction in the number of inflammatory cells in the BAL of both NOD1- and NOD2-deficient lean female mice, only NOD1 affected the respiratory function,^25^ which should be considered in future obesity studies.

Despite showing an increase of inflammatory cells in the BAL and lung tissue, we did not observe significant changes in IgE levels and pulmonary edema in NOD2-deficient mice during obesity. NOD2 can promote immune tolerance and balance innate immune responses.^32^^,^^33^ NOD2 can influence macrophages, other innate cells, and cytokines relevant to allergenic asthma.^34^ Although the mechanisms are not yet clear in the context of the lung and allergenic asthma, NOD2 can regulate the recruitment of eosinophils, independently of the presence of IgE. Hence, we hypothesize that in the absence of NOD2, IgE and pulmonary inflammation would be driven by alternative immune pathways that are still unknown. For edema, the absence of NOD2 may alter vascular permeability and the production of vasoactive cytokines, such as VEGF (vascular endothelial growth factor),^35^ reducing the ability to develop edema even in the presence of inflammation. Additionally, anti-inflammatory mechanisms may help prevent fluid accumulation in lung tissue, such as IL-10,^36^ which is elevated in obese NOD2-deficient mice. Although there is enough inflammation to induce the recruitment of eosinophils, the edema is also controlled by limiting fluid leakage. Evidence for microbiota-derived ligands such as MDP that can activate NOD2 and regulate the development and progression of pulmonary inflammation is an important future goal.

Microbiota is influenced by the diet.^37^^,^^38^^,^^39^^,^^40^ Diet-induced changes in the microbiota could alter the availability of peptidoglycans, where an increase in bioavailability of the NOD2 ligand MDP in non-obese female mice can enhance inflammation and worsen lung function.^41^ In our model, an HFD suppresses many characteristics of allergenic lung inflammation, and this requires NOD2. It is not yet clear how an HFD interacts with NOD2 and MDP to influence the complex factors that dictate lung inflammation versus tolerance. Increased type 2 responses and the activity of alveolar macrophages likely contribute to the exacerbated pulmonary inflammation seen in lean and obese female NOD2-deficient mice. IL-4 and IL-13 are cytokines produced by Th2 lymphocytes with key roles in lung remodeling by increasing subepithelial fibrosis and causing mucus hypersecretion due to the stimulation of hypertrophy and hyperplasia of goblet cells in the airways epithelial layer. IL-4 also promotes the eosinophils binding to blood vessel walls through increased expression of vascular cell adhesion molecule 1 (VCAM-1), contributing to the migration of these cells to the inflammation site.^42^ IL-5 is responsible for the maturation, growth, activation, and survival of eosinophils, the effector cell during allergy.^43^ IL-10 still has a controversial role, but its secretion by regulatory T and B cells suppresses the type 2 immune response and inflammation.^44^ CXCL1 has been shown *in vitro* to downregulate mast cell chemotaxis to airway smooth muscle,^45^ which inhibits the release of different inflammatory mediators and airway constrictors such as histamine and bioactive lipids.^46^

Resident lung macrophages polarize to the M2 phenotype in response to STAT6 and STAT3 activation by IL-4, IL-10, and IL-13.^47^ Reduced levels of nitrite and elevated levels of the enzyme arginase secreted in the BAL of obese NOD2-deficient mice support the hypothesis of IL-4-induced elevation of macrophage polarization to the M2 type, contributing to the observed inflammatory state. M2 macrophages are closely linked to the maintenance of immune homeostasis and allergic airway disease; however, its exact role in the pathophysiology of this disease is still poorly understood.^48^ Driving M2 polarization can mitigate inflammation and airway remodeling in OVA-exposed mice, similar to our results.^49^

Our study describes that NOD1 and NOD2 have distinct roles in allergic lung inflammation during diet-induced obesity. We highlight that NOD2 is required for lower allergen-induced inflammation in obese female mice. It is not yet clear how the intestinal or lung microbiota directly link to bacterial sensing and inflammation versus tolerance in the lung.

### Limitations of the study

Our study demonstrates that NOD2 provides protection from allergic lung inflammation in obese female mice. However, the cell type(s) responsible for NOD2-mediated protection from lung inflammation are not yet clear. Future work should also focus on defining a mechanism of action that may link specific postbiotics or functional units derived from the gut or lung microbiota to allergenic inflammation through bacterial sensors such as Nod2. Possible mechanisms that may be involved in the protective function of NOD2 include (1) regulation of immune tolerance versus pro-inflammatory signaling pathways, such as the NF-kB and MAPK pathways; (2) influence on the adaptive immune response through the activation of regulatory T cells (Tregs); and (3) alteration in gut barrier function or the gut microbiota. These mechanisms have been investigated by others in the context of NOD2’s role in reducing inflammation,^21^^,^^50^^,^^51^ and they may be necessary for pulmonary inflammation during obesity. The use of germ-free mice, targeted colonization of the microbiota, and conditional deletion of NOD2 in different cell types will be necessary to elucidate this question.

## Resource availability

### Lead contact

Further information and requests for resources and reagents should be directed to and will be fulfilled by the lead contact, Jonathan Schertzer (schertze@mcmaster.ca).

### Materials availability

This study did not generate new unique reagents.

### Data and code availability

• This paper analyzes existing, publicly available data. These accession numbers for the datasets are listed in the key resources table.
• This paper does not report the original code.
• Any additional information required to reanalyze the data reported in this paper is available from the lead contact upon request.

## Acknowledgments

The authors would like to thank The Sao Paulo Research Foundation (FAPESP, Brazil) for the financial support to carry out this study (grants 2019/03751-3 and 2021/09365-8). J.D.S. is a Canada Research Chair in metabolic inflammation. This work was supported by a discovery grant to J.D.S. from the 10.13039/501100000038Natural Sciences and Engineering Research Council of Canada (NSERC, RGPIN-2020-05707).

## Author contributions

Project design by G.F.A., J.D.S., and R.R.L.; execution of experiments by R.R.L., N.G.B., and H.F.; data analysis by R.R.L.; manuscript preparation by R.R.L., N.G.B., H.F., G.F.A., and J.D.S.

## Declaration of interests

The authors declare no competing interests.

## STAR★Methods

### Key resources table

**Table:** 

REAGENT or RESOURCE	SOURCE	IDENTIFIER
**Antibodies**		

CD206/MRC1 (E6T5J) XP Rabbit mAb	Cell Signaling	cat# 24595; RRID: AB_2892682
Goat anti-rabbit IgG (H + L) cross-adsorbed, Alexa Fluor 555	Thermo	cat# A-21428; RRID: AB_141784

**Chemicals, peptides, and recombinant proteins**		

Egg white albumin	Sigma	cat# A5503

**Critical commercial assays**		

Triglycerides assay	Stanbio	cat# 2200-430
Arginase Activity Assay Kit	Sigma	cat# MAK112
QIAzol Lysis Reagent	Quiagen	cat# 79306
DNAse I	Invitrogen	cat# 180068-015
SuperScript IV Reverse Transcriptase kit	Invitrogen	cat# 18090200
TaqMan Fast Advanced Master Mix	Thermo	cat# 4311806
Vector TrueVIEW Autofluorescence quenching kit	Vector	cat# SP-8400-15
Vectashield Antifade Mounting Medium with DAPI	Vector	cat# H-1200-10
TNF-α assay	BD	cat# 555268
IL-1β assay	R&D	cat# DY401
CXCL1 assay	R&D	cat# DY453
IL-4 assay	BD	cat# 555252
IL-5 assay	BD	cat# 555236
IL-10 assay	BD	cat# 555252
IL-6 assay	R&D	cat# DY406
TGF-β1 assay	R&D	cat# DY1679
IgE assay	BD	cat# 555248
IL-17a assay	R&D	cat# DY5390
IL-22 assay	R&D	cat# DY582

**Deposited data**		

NOD2 and allergic lung inflammation	Mendeley Data	https://doi.org/10.17632/97knh7zgkk.1

**Experimental models: Organisms/strains**		

C57Bl/6N	Charles River	RRID:IMSR_CRL:027
C57Bl/6J	Jackson Laboratory	RRID:IMSR_JAX:000664
NOD1−/−	Denou et al.^21^; Schertzer et al.^22^	N/A
NOD2−/−	Denou et al.^21^ , Schertzer et al.^22^	N/A

**Oligonucleotides**		

*Actb*	Thermo	cat# mm02619580
*Arg1*	Thermo	cat# mm01190441

**Software and algorithms**		

ImageJ 1.46r	NIH	RRID:SCR_003070
ImagePro Plus	Media Cybernetics	RRID:SCR_007369
GraphPad Prism software, version 9	GraphPad Software	RRID:SCR_002798

**Other**		

High fat diet	Envigo	cat# 8640
Chow diet	Research Diets Inc.	cat# D12492

### Experimental model and study participant details

#### Animals

The procedures were approved by the McMaster University Animal Research Ethics Board (AREB) in accordance with the guidelines of the Canadian Council of Animal Care. All mice were kept on a 12-h light/dark cycle in collective cages with 4–5 mice. We used female Nod1^−/−^ and Nod2^−/−^ whole body knockout mice at the Central Animal Facility (CAF) at McMaster University. Nod1^−/−^ have been backcrossed more than 12 generations to C57Bl/6N mice, hence C57Bl/6N (Strain code cat# 027, RRID:IMSR_CRL:027, Charles River) mice were used as controls (WT) for Nod1^−/−^ mice. Nod2^−/−^ mice have been backcrossed more than 10 generations to C57Bl/6J mice, hence C57Bl/6J (cat# 000664, RRID:IMSR_JAX:000664, Jackson Laboratory) were used as controls (WT) for Nod2^−/−^ mice. These mice and their respective background controls have been used in a previous study published by our group.^52^

#### Experimental design

Animals were 8 weeks old at the beginning of the experimental procedures. Female Nod1^−/−^ and Nod2^−/−^, C57Bl/6N and C57Bl/6J mice were fed with either standard chow (SC) (17% Kcal of fat, Envigo, Indianapolis, USA, cat# 8640) or a high-fat diet (HFD) (60% Kcal of fat and 7.24% Kcal from sucrose, provided by Research Diets Inc., New Brunswick, USA, cat# D12492) supplemented with commercially available whole roasted peanut butter (68.8% Kcal of fat and 0% Kcal of sucrose without the addition of colorings, sweeteners and flavorings, provided by Nut, Marília, Brazil) for 10 weeks. Egg white albumin (OVA Grade V) (Sigma cat# A5503) was used as an allergenic agent initiated 17 days before the endpoint. First, sensitization was performed on day 0 by an intraperitoneal administration of 50 μg of OVA dissolved in 2 mg of aluminum hydroxide (in 400 μL of sterile saline). A booster dose of OVA was provided on day 7 and was administered the same way as the initial dose of OVA. The challenge doses of OVA were administered as 5% OVA (in sterile saline) by aerosol for 25 min on days 14, 15 and 16 of the experiment, totaling 3 challenges after 2 sensitization doses of OVA. The aerosol was administered through an ultrasonic nebulizer (Power Neb Ultra Nebulizer cat# 18081) coupled to a 28 cm^2^ acrylic chamber.^4^ Mice were sacrificed under anesthesia with an intraperitoneal dose of ketamine/xylazine (100/10 mg/kg) followed by cervical dislocation.

### Method details

#### Bronchoalveolar lavage (BAL) collection and ponderal and metabolic endpoints

Body mass was measured, and adiposity was measured with Time-Domain Nuclear Magnetic Resonance (TD-NMR), MINISPEC LF90II (Bruker, Massachusetts, USA). For BAL collection, the trachea was cannulated with a catheter (24G) followed by three flushes with 500 μL of sterile PBS were sequentially performed into the lung cavity and the combined volume was centrifuged (1000 × g, 10 min, 4°C) to obtain the cell fraction and the supernatant.^53^ Supernatants were stored at −80°C for further analysis. Cell pellets were reconstituted in 1 mL of sterile PBS and used for total cell count in a Neubauer chamber (in Turk’s solution), and differential cell counts were conducted on cytospin-mounted slides stained with Wright-Giemsa to determine cell population. Blood was obtained by extracorporeal cardiac puncture with the aid of a heparinized syringe attached to a 26G needle. Blood glucose was measured with a glucometer (Medisure Empower SN).

#### Liver triglycerides levels

Liver fragments (100 mg) were processed in Chloroform/Methanol solution (2:1) using an automatic homogenizer (MP FastPrep-24) and vigorously agitated overnight (4°C). Samples were diluted with 200 μL of 0.6% NaCl and the organic phase (chloroform containing lipids) was carefully collected, transferred to a new tube and allowed to dry at room temperature. The tube walls were washed with isopropanol to recover dried lipids that were next quantified using a colorimetric commercial kit (Stanbio cat# 2200-430).^54^

#### Pulmonary edema

The right lower lung lobe was excised and massed. Samples were then dried (50°C for 5 days) and the mass of the dried tissue was assessed. Edema was expressed as the ratio of the wet/dry sample mass according to method previously published by Matsuyama and collaborators.^55^

#### Histological analysis

The entire single lobe of the left lung and the liver left lobe were excised and fixed in PBS containing 4% paraformaldehyde for 3 days. After the fixation, samples were washed 3 times with PBS to remove the excess of paraformaldehyde and immersed in 70% ethanol followed by paraffin embedding (EG1160 Leica, Nussloch, Germany) as described.^56^ 5 μm sections were obtained with an HM325 microtome (Thermo Scientific, Waltham, USA) and immobilized on a microscope slide. All slides were oven dried at 37°C for 24 h before staining. Tissue hydration, staining (Hematoxylin/Eosin, Luna, Masson’s Trichrome and Periodic Schiff Acid/Alcian blue), diaphanization, and Permount mounting were performed, as described.^57^ Histological images were obtained using a microscope H600L (Nikon, Tokyo, Japan) with a magnification of 200× coupled to the DS-Qi2 camera (Nikon, Tokyo, Japan). Histomorphometric measurements were determined using ImageJ 1.46r (NIH, RRID:SCR_003070) and ImagePro Plus (Media Cybernetics, RRID:SCR_007369) software.

#### Pulmonary leukocyte infiltrate quantification

Five random images of bronchioles from each section stained with H&E were analyzed using ImageJ 1.46r software (NIH, RRID:SCR_003070). The leukocyte infiltrate present in the peri bronchoalveolar and perivascular space was manually defined as a single area using the “Freehand selections” tool. The size of the area (in μm^2^) was subsequently determined according to the previous calibration of the scale of each image, providing the parameter “Leukocyte infiltrate area”. The determination of eosinophils and non-eosinophils were performed by differential counting using Luna staining, where 100 leukocytes counted in a zigzag pattern were manually identified in the peri bronchoalveolar and perivascular space, providing the percentage of both cell types. The area of eosinophils and non-eosinophils was subsequently estimated by deconvolution the percentage of these cells under the infiltrate area.^53^ Lung leukocyte quantification was confirmed by measuring the enzymatic activity of the eosinophilic peroxidase (EPO), as described.^58^

#### Histological quantification of mucus

Five random images of bronchioles from each section stained with PAS/Alcian blue were analyzed using ImagePro Plus software (Media Cybernetics, RRID:SCR_007369). The colors purple-magenta (PAS, neutral mucins) and turquoise blue (Alcian blue, acid mucins) were automatically detected by the software and expressed as the percentage of the tissue section area.^4^^,^^53^^,^^59^ The tissue section area was calculated by the software and was defined as the picture area subtracted from the bronchioles and vessels lumen area (empty bronchioles and vessels lumen area were automatically outlined by the software). Goblet cell hypertrophy was measured similarly to the quantification of leukocyte infiltrate (in μm^2^). Goblet cell hyperplasia was determined manually in random bronchioles using a 100 μm linear line.^53^

#### Lung remodeling

Pulmonary fibrosis and tracheal stenosis were measured by quantifying levels of hydroxyproline.^60^ Tissue samples of the lung and trachea were dehydrated in an oven at 50°C for 5 consecutive days, then dissociated in 5 M HCl (20 mg/mL) and subjected to acid hydrolysis in an autoclave at 121°C for 45 min. After cooling, the hydrolysate was neutralized with 1 M NaOH (1:5). 100 μL of neutralized hydrolysate was transferred to a 96-well plate, where it received 100 μL of oxidation solution (chloramine T in ethanol). After 5 min under agitation, 100 μL of Ehrlich’s Solution were added and the plate was then incubated at 60°C for 45 min. Hydroxyproline was measured spectrophotometrically at 570 nm and compared to a collagen standard (Sigma cat# C9879). Collagen was also determined histologically, similarly to what was described in the determination of mucus, by the dark blue staining from Masson’s Trichrome.

#### Arginase activity in BAL

Arginase activity in BAL supernatants was assessed using the Arginase Activity Assay Kit (Sigma cat# MAK112) following the manufacturer’s instructions.

#### Quantitative PCR

RNA was extracted from the lung tissue using QIAzol Lysis Reagent (Qiagen cat# 79306) in an automatic homogenizer (MP FastPrep-24). RNA purity was subsequently determined by the absorbance ratio (260 nm/280 nm), where samples in the range 1.8–2.1 were considered acceptable. cDNA was synthesized from 1 μg of total RNA, previously treated with DNAse I (Invitrogen cat# 18068-015), using the SuperScript IV Reverse Transcriptase kit (Invitrogen cat# 18090200). Quantitative PCR was performed in Rotor-Gene Q (Qiagen, Ohio, USA) using TaqMan Fast Advanced Master Mix (Thermo cat# 4311806) and 40 ng of cDNA per reaction. The reactions consisted in 50 cycles of 95°C for 10 s and 58°C for 45 s. The quantification of each gene was determined by the 2^−ΔΔCt^, represented by the difference in expression between the target gene and the endogenous control gene (*Actb*). Assay IDs for the primers used were *Actb* (mm02619580) and *Arg1* (mm01190441).

#### Immunofluorescence

Sections of lung embedded in paraffin were hydrated and had the epitopes recovered in Tris-EDTA buffer using high pressure and a temperature of 203°F. The tissue was blocked in PBS-T with 5% BSA for 60 min and incubated overnight with the primary antibody CD206/MRC1 (E6T5J) XP Rabbit mAb (Cell Signaling cat# 24595; RRID: AB_2892682 1:200). After 3 washes with PBS-T, the tissue was incubated in the dark for 1 h with the secondary antibody goat anti-rabbit IgG (H + L) cross-adsorbed, Alexa Fluor 555 (Thermo cat# A-21428; RRID: AB_141784 1:250) and treated with the Vector TrueVIEW Autofluorescence quenching kit (Vector cat# SP-8400-15) to reduce lung autofluorescence. Slides were mounted with Vectashield Antifade Mounting Medium with DAPI (Vector cat# H-1200-10) and sealed with nail polish before imaging. Random images were obtained using the appropriate filters (DAPI and FITC) and had the fluorescence (integrated density) determined by ImageJ 1.46r software (NIH, RRID:SCR_003070).

#### Cytokine levels

The levels of TNF-α (BD cat# 555268), IL-1β (R&D cat# DY401), CXCL1 (R&D cat# DY453), IL-4 (BD cat# 555252), IL-5 (BD cat# 555236), IL-10 (BD cat# 555252), IL-6 (R&D cat# DY406), TGF-β1 (R&D cat# DY1679), IgE (BD cat# 555248), IL-17a (R&D cat# DY5390) and IL-22 (R&D cat# DY582) were measured in BAL supernatant or in plasma using commercially available ELISA kits following the manufacturer’s instructions.

### Quantification and statistical analysis

Statistical analysis was performed using the GraphPad Prism software, version 9 (RRID:SCR_002798) using Unpaired Student’s *t* test. To assess outliers, the results were also processed by the ROUT test (Q = 1%). All values with *p* < 0.05 were considered statistically significant.
